# Angiogenesis-related lncRNAs index: A predictor for CESC prognosis, immunotherapy efficacy, and chemosensitivity

**DOI:** 10.7150/jca.94332

**Published:** 2024-04-08

**Authors:** Xueyuan Huang, Guangming Yi, Jiayu Xu, Siqi Gou, Haiqing Chen, Xiaoyan Chen, Xiaomin Quan, Linjia Xie, Alexander Tobias Teichmann, Guanhu Yang, Hao Chi, Qin Wang

**Affiliations:** 1 Clinical Medical College, Southwest Medical University, Luzhou 646000, China.; 2 Department of Oncology, The Third Hospital of Mianyang (Sichuan Mental Health Center), Mianyang, Sichuan 621000, China.; 3 School of Science, Minzu University of China, Beijing 100081, China.; 4 Department of Obstetrics and Gynecology, The First Affiliated Hospital of Wenzhou Medical University, China.; 5 Beijing University of Chinese Medicine, 100029, Beijing, China.; 6 Department of Oncology, Beijing University of Chinese Medicine second affiliated Dong Fang hospital, 100078, Beijing, China.; 7 Sichuan Provincial Center for Gynecology and Breast Diseases (Gynecology), Affiliated Hospital of Southwest Medical University, Luzhou 646000, China.; 8 Department of Specialty Medicine, Ohio University, Athens 45701, OH, United States.

**Keywords:** CESC, LncRNAs, Angiogenesis, Immunotherapy, Chemotherapy, Prognostic signature

## Abstract

Cervical squamous cell carcinoma and endocervical adenocarcinoma (CESC) is a common gynecologic tumor and patients with advanced and recurrent disease usually have a poor clinical outcome. Angiogenesis is involved in the biological processes of tumors and can promote tumor growth and invasion. In this paper, we created a signature for predicting prognosis based on angiogenesis-related lncRNAs (ARLs). This provides a prospective direction for enhancing the efficacy of immunotherapy in CESC patients. We screened seven OS-related ARLs by univariate and multivariate regression analyses and Lasso analysis and developed a prognostic signature at the same time. Then, we performed an internal validation in the TCGA-CESC cohort to increase the precision of the study. In addition, we performed a series of analyses based on ARLs, including immune cell infiltration, immune function, immune checkpoint, tumor mutation load, and drug sensitivity analysis. Our created signature based on ARLs can effectively predict the prognosis of CESC patients. To strengthen the prediction accuracy of the signature, we built a nomogram by combining signature and clinical features.

## Introduction

Cervical cancer is a common gynecologic malignancy that has a significant impact on women's health and is a major contributor to cancer-related mortality in women. It is estimated that approximately 500,000 new cases of cervical cancer are diagnosed annually, with more than 300,000 women succumbing to the disease 1. The main treatments currently used for cervical cancer include radiation and chemotherapy, but not everyone who receives these therapies has significant outcomes, and sometimes even serious adverse effects 2. The key factors affecting the prognosis of patients with cervical cancer are whether the tumor has metastasized and whether the patient receives effective adjuvant therapy 3. The early stage of cervical cancer is usually asymptomatic or non-specific, resulting in many patients not being diagnosed at an early stage 4. Patients diagnosed at the early stages of the disease can have a good prognosis by undergoing radical surgery and adjuvant therapy, but patients with advanced tumors, metastases, or recurrence often have a poor prognosis 5, and patients with advanced cervical cancer urgently need therapies to improve survival. Recent advances in immunotherapy have been explored as a potential treatment for cervical cancer, yet have not been able to improve the prognosis of patients 1. Molecular biomarker discovery can help to predict treatment outcomes and survival while differentiating between high- and low-risk patients 1. The discovery of new diagnostic biomarkers can facilitate the development of precision medicine and is also very beneficial for improving treatment and developing new immune-targeted therapies. Consequently, there is an urgent requirement to find more sensitive biomarkers for early diagnosis, treatment, and prognosis of cervical cancer.

Angiogenesis is a highly coordinated process by which existing blood vessels generate new blood vessels 6. Tumor angiogenesis provides oxygen and nutrients to the tumor and can provide access to tumor metastasis 7. Abnormal regulation of angiogenesis is a fundamental process in cancer invasion and metastasis and is an important factor in controlling cancer progression 8. Anti-angiogenic and pro-angiogenic factors are closely related to angiogenic homeostasis. When both are in equilibrium, endothelial cells are nonproliferative and do not lead to angiogenesis. When pro-angiogenic factors are dominant, they lead to angiogenesis, a process defined as an "angiogenic switch" in tumors 9. Judah Folkman believes that tumor development requires the initiation of angiogenesis 10, based on the fact that extensive vascularization is observed in rapidly growing tumors, but not in dormant tumors. Many anti-angiogenic drugs have been approved for use in cancer treatment. However, the diversity of compensatory mechanisms for vascular remodeling has led to a decrease in the anti-angiogenic activity of anti-angiogenic drugs based on a single mechanism 11. For patients with recurrent and advanced cervical cancer, the use of antiangiogenic drugs did not significantly improve patient prognosis and did not achieve satisfactory clinical outcomes 12.

Long non-coding RNAs (lncRNAs) are specified as RNA transcripts that are over 200 nucleotides in length and do not code for proteins, and are implicated in a range of major biological functions and pathological processes, including proliferation, apoptosis, metastasis and migration 13-15. According to studies, aberrant expression of lncRNAs interacting with epigenetic modifiers can disrupt epigenetics leading to cellular dysregulation, malignant transformation and altered gene function 16, 17. It has been suggested that IncRNAs may be involved in the development and progression of tumors, such as accelerating tumor growth, metastasis, immune escape, regulating energy metabolism and promoting angiogenesis. Aberrant expression of lncRNAs has been detected in various cancers, and lncRNAs can function as tumor suppressors or influence the expression of associated genes 18-20. Several studies have shown aberrant lncRNA expression in cervical cancer 2. CCHE1, a lncRNA positioned in the intergenic region of chromosome 10, is able to bind to mRNA to upregulate the expression of nuclear antigen in proliferating cells, thus promoting the proliferation of cervical cancer cells 21. LINC01305 has increased expression in cervical cancer cells, resulting in reduced patient survival. This lncRNA has been demonstrated to facilitate the progression of cervical cancer through KHSRP and can release exosomes to participate in the stemness of cancerous tissues 22. It has been suggested that many IncRNAs may serve as potential biomarkers and therapeutic targets for cancer diagnosis and treatment 23. It has been demonstrated that numerous IncRNAs are closely related to the progression of cervical cancer, but ARLs have been less studied in cervical cancer. Therefore, exploring the role of angiogenesis-related lncRNAs as tumor biomarkers in the treatment and prognosis of cervical cancer is very promising and can facilitate the development of precision medicine in targeted immunotherapy of tumors.

Consequently, we explored the potential role of ARLs on the survival of CESC patients. In our research, we screened seven reliable ARLs based on CESC samples from TCGA database for constructing risk prognosis Signature and forming risk score. Subsequently, we further investigated the correlation between ARLs and immune infiltration, immunotherapy, tumor mutational load and targeted drug sensitivity. We hope that our study will uncover molecular biomarkers associated with the prognosis of cervical cancer patients and develop novel techniques to improve treatment strategies to enhance patient prognosis.

## Methods

### Patient Data Sources

We collected clinical and transcriptomic information from 307 patients with CESC from the TCGA database (https://portal.gdc.cancer.gov/) (downloaded on October 3, 2022). The Strawberry Perl software was utilized to differentiate mRNA from lncRNA from transcriptomic data. Follow-up studies were conducted mainly based on lncRNA. Clinical information included patient's age, gender, grade, TMN stage, and overall survival. we randomly divided the CESC patients into training and test groups at the ratio of 8:2. We obtained 36 Angiogenesis-related genes (ARGs), and the information of these genes is shown in **Table S1**.

### Construction and Validation of the Prognostic Model

We first used the R package "limma" to obtain differentially expressed genes, and then we screened and obtained ARLs by investigating the relationship between ARGs and lncRNAs expression (screening criteria: correlation coefficient > 0.4, p-value < 0.001), in which ARLs satisfying |log2 fold change| > 2 and a false discovery rate (FDR) < 0.05 were considered as differentially expressed ARLs. We then performed univariate regression analysis and Lasso regression analysis using the R package "glmnet" to identify prognosis-related ARLs, and multivariate regression analysis to identify modeled genes and their coefficients. The risk score for each patient was calculated as follows: Risk Score = CoeflncRNA_1_ × ExpressionlncRNA_1_ + CoeflncRNA_2_ × ExpressionlncRNA_2_ + CoeflncRNA_n_ × ExpressionlncRNA_n_. We used time-dependent ROC curves to validate the established model. To make the validation results more reliable, we also show the time-dependent ROC curves for the training and test groups for internal validation. We divided CESC patients into two groups according to riskscore, low risk and high risk, and subsequently performed Kaplan-Meier analysis on these two groups and compared their overall survival.

### Construction of Nomogram

In order to verify whether the risk score can be used as an independent prognostic factor, we performed univariate Cox and multivariate Cox analyses, and drew forest plots based on the results. We then used the R package "rms" to construct a nomogram containing risk scores and other clinicopathological characteristics to predict the prognosis of survival at 1, 3, and 5 years for the CESC samples in the TCGA database.

### Functional Enrichment Analysis

We performed Gene Ontology (GO) enrichment analyses of the differentially expressed genes associated with the seven ARLs using the R package "ClusterProfifiler" and visualized the results with the R package "circlize " to visualize the results. We used the R package "GSVA" and "c2.cp.kegg.v7.4.symbols.gmt" from MSigDB for GSVA enrichment analysis, and the R package "heatmap" for heat map. heatmap" to draw heat maps.

### Immunological Analysis

We used seven algorithms, XCELL, TIMER, QUANTISEQ, MCPCOUNT, EPIC, CIBERSORT, and CIBERSORT-ABS, to score immune infiltration. We then performed Spearman correlation analysis to explore the immune cell infiltration and risk scores, and showed the immune cell infiltration between high and low risk groups with violin plots. We identified 20 immune checkpoint-associated genes for analysis by Auslander's study 24 to compare differences in immune checkpoint expression between high- and low-risk groups. We used ssGSEA analysis to examine the differences in immune function between the high- and low-risk groups.

### Tumor Mutation Load Analysis

We obtained the tumor mutation load (TMB) of CESC patients from the TCGA database (https://portal.gdc.cancer.gov/) (downloaded on October 3, 2022). We used Strawberry Perl to analyze the TMB and used the R package "maftool" in R software to analyze the 15 genes with the highest mutation frequency in the CESC samples. Then, we divided the patients into two groups by TMB values: high TMB and low TMB. We also combined risk score and TMB to classify patients into four groups: H-TMB+high risk, H-TMB+low risk, L-TMB+high risk, and L-TMB+low risk. We completed the survival analysis of these four groups by log-rank test.

### Drug Sensitivity Analysis

We assessed the drug sensitivity of patients in the high and low risk groups based on the half-maximal inhibitory concentration (IC50) of the Genomics of Drug Sensitivity to Cancer (GDSC) (https://www.cancerrxgene.org/) for each CESC patient. We used the R package "pRRophetic" in this process.

### Statistical Analysis

We used R software (version 4.2.1) and Strawberry Perl (version 5.30.0) to perform the bioinformatics analysis for this study. In general, we considered *P*<0.05 to be statistically significant and FDR < 0.05 is statistically significant. The data are presented as means ± standard deviation (SD) from three independent experiments and analyzed by analysis of variance (ANOVA). Statistical significance was set at *P* < 0.05. "*" Means *P* < 0.05, "**"Means *P* <0.01, "***" Means *P* < 0.001.

## Results

### Acquisition of Candidate ARLs in CESC

**Figure 1:**
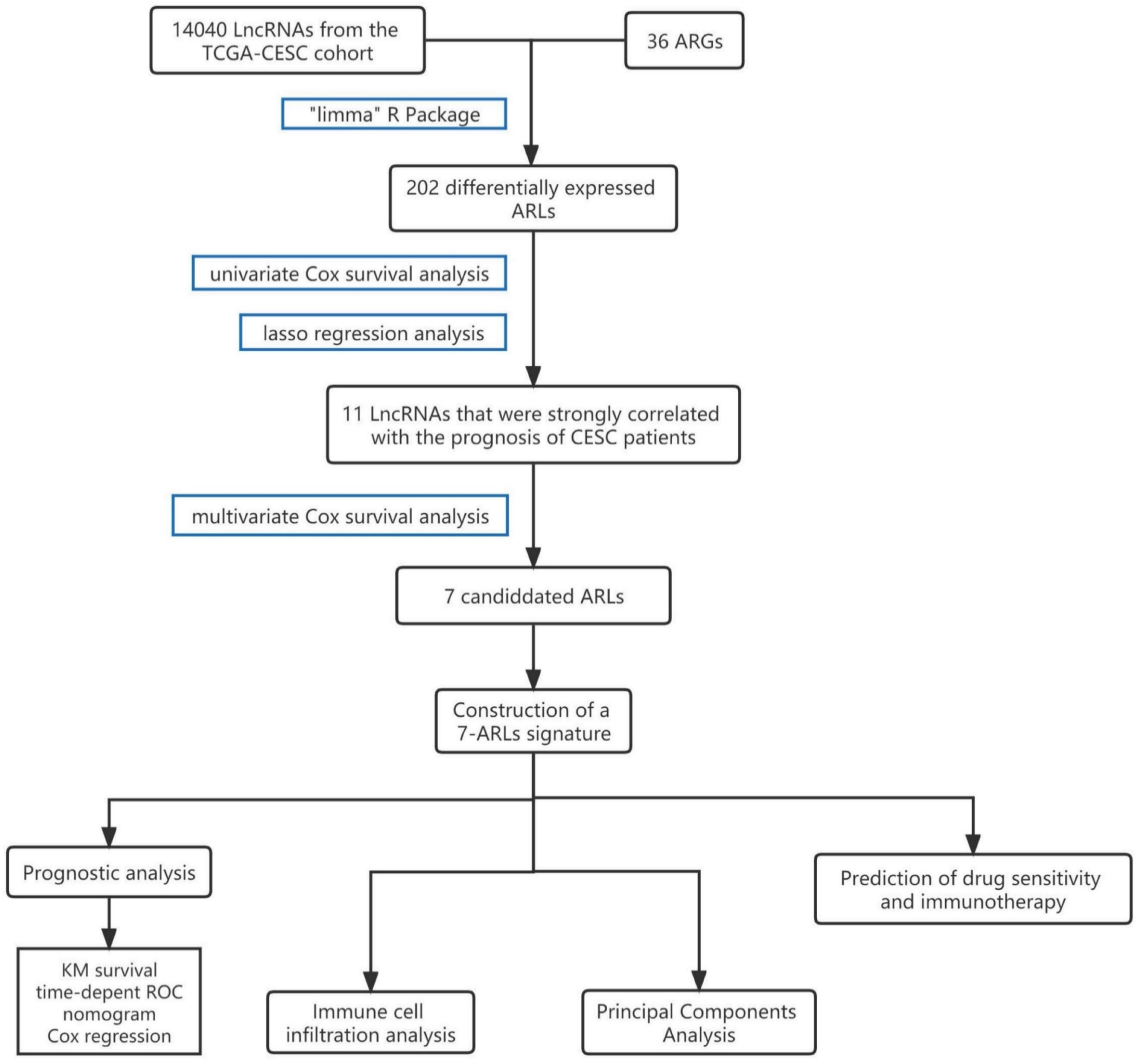
A flowchart outlining the study's primary ideas.

**Figure 2:**
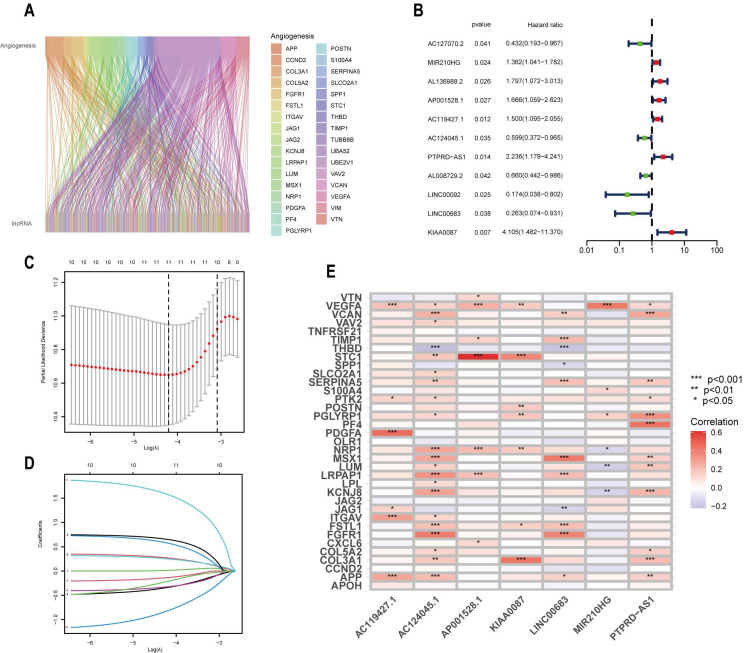
Screening of candidate ARLs. (A) Sankey diagram showing the co-expression relationship between ARLs and ARGs. (B) Prognosis of ARLs evaluated using univariate Cox regression analysis. (C) Adjusted parameter selection in Lasso analysis by tenfold cross-validation. (D) Lasso coefficient graph. (E) Heat map presenting the correlation of candidate ARLs and ARGs.

The flowchart illustrates the main idea and content of this study **(Figure 1)**. We obtained 202 ARLs by Pearson correlation analysis with screening conditions of correlation coefficients >0.4 and p < 0.001** (Table S3)**. And the co-expression relationship between ARGs and ARLs was demonstrated by plotting the sankey diagram **(Figure 2A)**.

### Construction of Prognostic Signature of ARLs

In order to enhance patient prognosis and further the development of precision medicine, we created a prognostic model relying on ARLs in CESCC patients. Initially, we integrated the expression of ARLs with the clinical survival data from the TCGA-CESC cohort. We obtained 11 OS-related ARLs by univariate regression analysis (P < 0.05)** (Figure 2B)** and lasso regression analysis, and analyzed their regression coefficients and cross-validation trends, the number of LncRNAs engaged in model construction was determined by taking the lowest point of the cross-validation plot of eleven **(Figure 2C,D)**. Finally, we identified 7 candidate ARLs by multifactorial regression analysis, namely MIR210HG, AP001528.1, AC119427.1, AC124045.1, PTPRD-AS1, LINC00683 and KIAA0087. A prognostic prediction model was developed based on these 7 ARLs and this formula was obtained by weighting the regression coefficients on the identified ARLs: Risk Score=(0.3546×ExpressionMIR210HG)+(0.7189×ExpressionAP001528.1)+(0.3485×ExpressionAC119427.1)+(-0.5958×ExpressionAC124045.1)+(0.6929×ExpressionPTPRD-AS1)+(-1.7296×ExpressionLINC00683)+(2.2408×ExpressionKIAA0087). In addition, in order to understand the correlation between the obtained LncRNAs and ARGs, we also conducted co-expression analysis of ARGs and ARLs (Figure 2E), based on the results we can see VEGFA, NRP1 and APP have more correlation with OS-related ARLs compared to other genes.

### Validation of the Prognostic Value and Signature Independence of the Model

**Figure 3:**
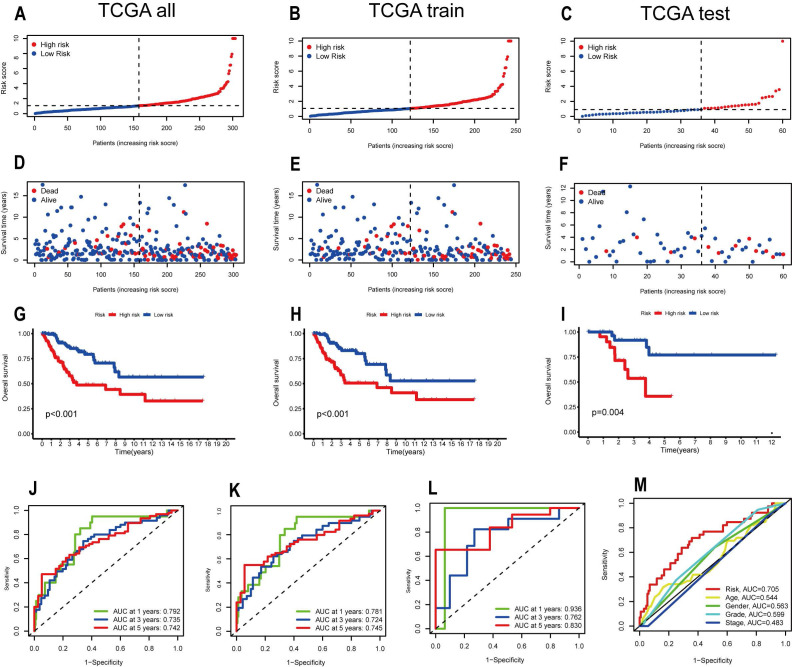
Verification of the accuracy of the ARLs signature in predicting prognosis. Distribution of risk scores between high and low risk groups in (A) TCGA all, (B) TCGA train, (C) TCGA test cohorts. Survival status between low and high risk groups in the (D) TCGA all, (E) TCGA train, (F) TCGA test cohorts. Survival Curve between low and high risk groups in the (G) TCGA all, (H)TCGA train, (I) TCGA test cohorts. Time-dependent ROC curves are demonstrated in (J) for all patient groups and (K) for the training group (L) for the test group. (M) Multi-index ROC analysis.

To verify whether the Signature constructed by 7 OS-related ARLs has good prognostic value for CESC patients, we analyzed and evaluated the model. We divided CESC patients into high-risk and low-risk groups according to their risk scores for subsequent validation of the model. First, we ranked the CESC patients by risk score in the All, Train, and Test groups and plotted the patients' survival status as scatter plots, and the same trend of increasing mortality with increasing risk score was observed in all three groups** (Figure 3A-F)**. Also, we used Kaplan-Meier analysis. and in the All group it could be seen that the prognosis and clinical outcome of patients in the high-risk group were much worse than those in the low-risk group (p < 0.001) **(Figure 3G)**. In the time-dependent ROC curves, the AUCs at 1,3 and 5 years were 0.792, 0.735 and 0.742, respectively **(Figure 3J)**. survival curves in the Train group also showed significantly better clinical outcomes for patients in the low-risk group than in the high-risk group (P < 0.001)** (Figure 3H)**. In the time-dependent ROC curves, the AUCs at 1,3 and 5 years were 0.781, 0.724 and 0.745, respectively **(Figure 3k)**. The Kaplan-Meier analysis for the Test group led to the same conclusion as the previous two groups (p=0.004)** (Figure 3I)**. The ROC curves for the Test group showed AUCs of 0.936, 0.762 and 0.830 **(Figure 3L)**. The ROC curves for all three groups showed high specificity and accuracy of this model. We also performed a Multi-index ROC analysis, and from the results it is clear that risk scores have an advantage over traditional clinicopathological characteristics in predicting the prognosis of CESC patients (Risk, AUC=0.705).

### Principal Components Analysis

**Figure 4:**
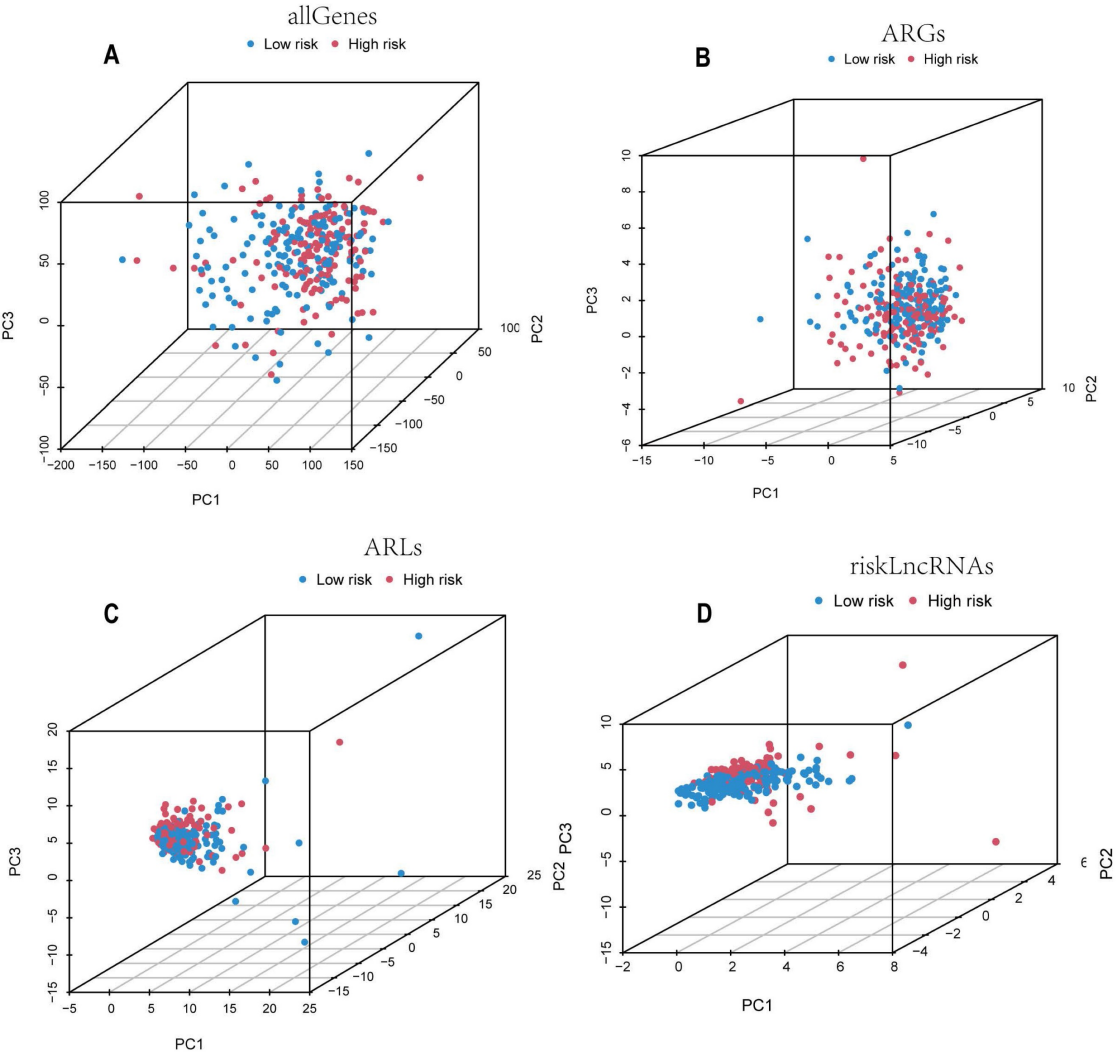
Candidate ARLs can better differentiate between high-risk and low-risk cohorts. PCA plots of the all genes (A), ARGs (B), ARLs (C), and candidate ARLs (D).

By performing principal components analysis (PCA) analysis on all genes, all ARGs, all ARLs and the candidate lncRNAs **(Figure 4A-D)**, we could observe whether they could clearly distinguish the patients in the low-risk and high-risk groups. The results of Figure 4D's study demonstrate that the model we developed is remarkably predictive because the lncRNAs used to build the model are able to discriminate patients in the low-risk group from those in the high-risk group more effectively than the other three.

### Association between Clinicopathological Features and Risk Scores

**Figure 5:**
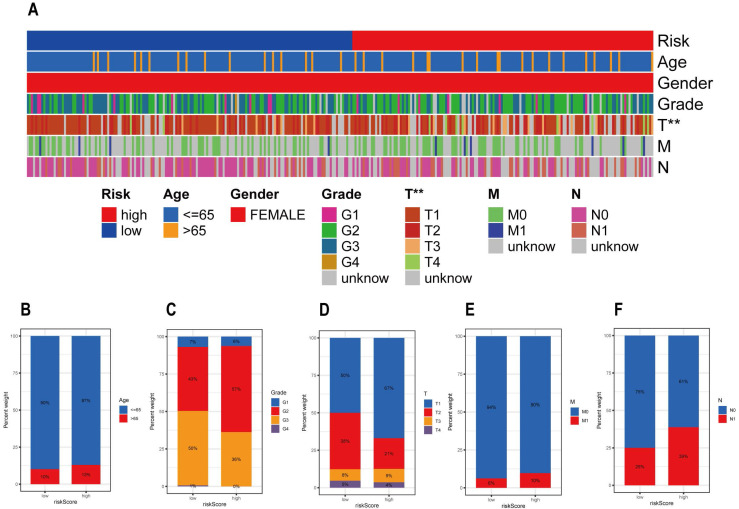
Association between risk scores and clinicopathological characteristics. (A) Heat map illustrating the proportion of risk scores and clinicopathological features in the TCGE-CESC cohort. Differences in the number of patients with different clinical characteristics in the high- and low-risk groups. These clinical characteristics include (B) age, (C) grade, (D) T stage, (E) M stage, and (F) N stage.

**Figure 5A** shows the rickscore, age, gender, grade, T stage, M stage and N stage of the CESC sample in the TCGA database. Figure 5B-F details the proportion of patients with different clinicopathological characteristics in the high and low risk groups. By analyzing the correlation between risk scores and clinicopathologic features, we can conclude that the frequencies of the four clinicopathologic features, grade, T stage, M stage and N stage, differed more significantly between the high and low risk groups.

### Survival Analysis of Groups with Different Clinicopathological Characteristics

**Figure 6:**
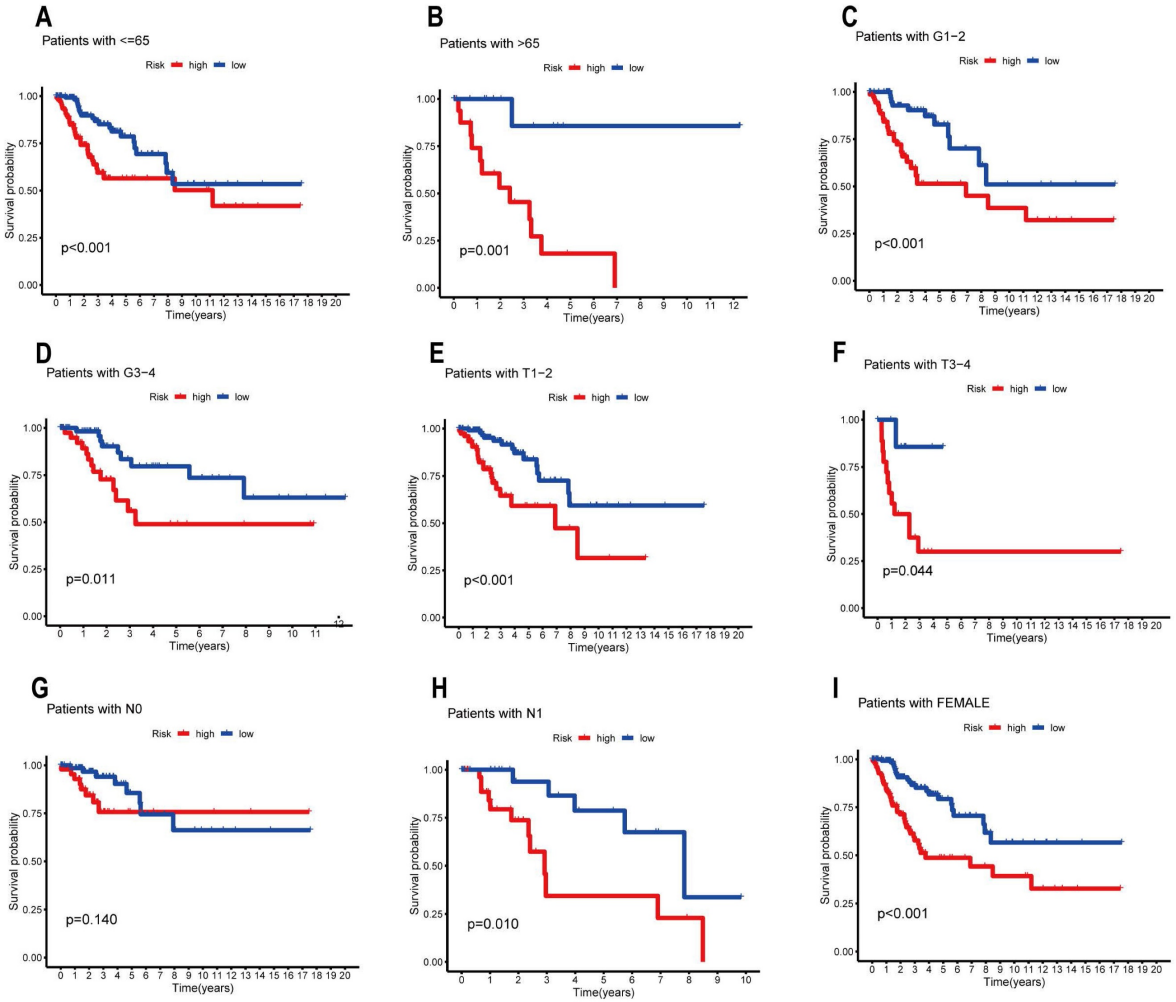
Prognostic efficacy of the ARL risk model for overall survival of different subtypes in the TCGA-PAAD cohort, (A) age≤65, (B) age>65, (C) grades I-II, (D) grades III-IV, (E) T1-T2, (F) T3-T4, (G) N0, (H) N1 and (I) female, respectively.

We used the R package "survival" and "survminer" to analyze the survival of subgroups with different clinicopathological characteristics in order to further understand whether the prognosis of patients in the high and low risk groups differed among the subgroups. The samples were separated into various subgroups according to age (65 years old, >65 years old), grade (G1-2, G3-4), T stage (T1-2, T3-4), N stage (N1, N2), and gender (only female). Subsequently, we assessed survival in subgroups and plotted the corresponding Kaplan-Meier curves** (Figure 6)**. Overall survival was substantially worse in the high-risk group than in the low-risk group in all remaining clinical categories, with the exception of age = 65 and N0 stage. These findings suggest that a risk-prognosis model based on seven ARLs associated with prognosis can predict the prognosis of different clinical subgroups of CESC patients more accurately.

### Development of Nomogram Based on Clinicopathological Features

**Figure 7:**
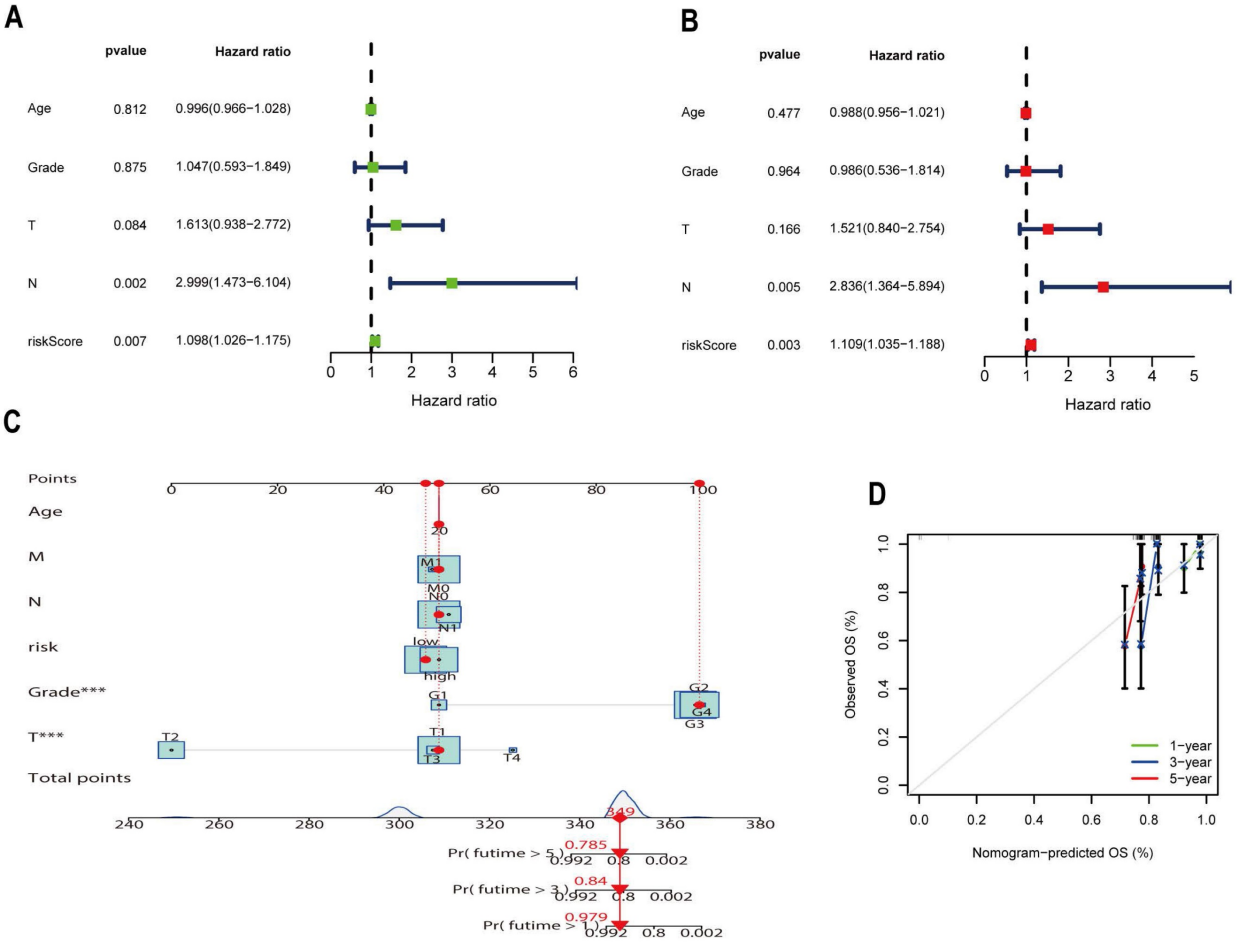
A nomogram constructed by combining risk scores and clinical characteristics. (A) Univariate and (B) Multivariate analysis of risk scores and multiple clinical characteristics. (C) Nomogram predicting overall survival of CESC patients at 1,3 and 5 years. (D) The calibration curve of the created nomogram.

To clarify whether the risk-prognosis model and different clinicopathological characteristics could be used as independent prognostic indicators for patients with CESC, we performed univariate and multifactorial Cox regression analyses of the TCGA-CESC cohort. In terms of the results of the univariate Cox regression analysis, the risk score was a significant prognostic predictor (P=0.007, HR=1.098, 95CI=1.026-1.175) **(Figure 7A)**. Follow-up multifactorial Cox regression analysis further validated that the risk score had superior independent predictive power of prognosis (P=0.003, HR=1.109, 95CI=1.035-1.188) **(Figure 7B)**. We used the patient's age, T stage, M stage, N stage, grade, and riskscore to construct the nomogram **(Figure 7C)**, which can be used to predict the survival of CESC patients at 1, 3, and 5 years, and to quantify the patient's OS using the nomogram. The nomogram combines the prognostic model developed by the 7 ARLs with other clinicopathological features, which helps to improve the model's deficiencies and thus improves the prognostic accuracy. The 1,3 and 5-year calibration curves of the column line graph likewise illustrate good agreement between predicted and observed outcomes, demonstrating its powerful ability to predict prognosis** (Figure 7D)**.

### Functional Enrichment Analysis

**Figure 8:**
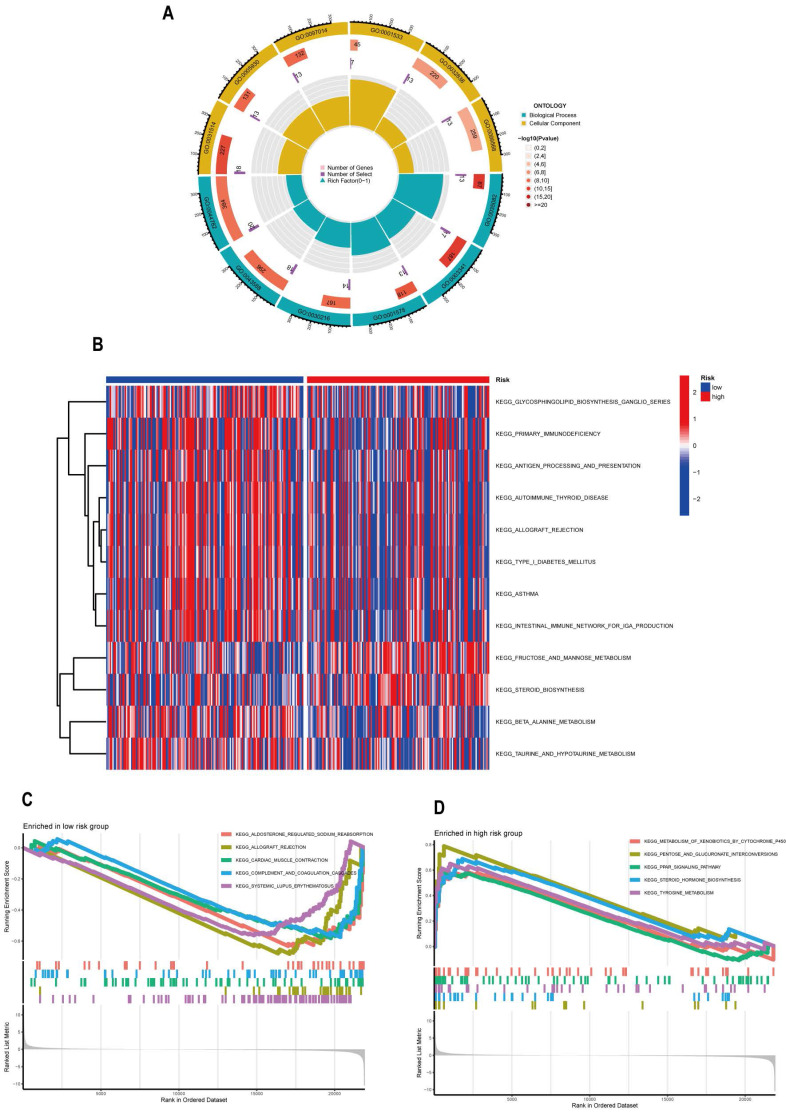
Functional enrichment analysis of ARLs in the TCGA-CESC cohort. (A) GO enrichment analysis. (B) GSVA analysis between the high-risk and low-risk group with Kyoto Encyclopedia of Genes and Genomes (KEGG). GSVA analysis between the (C) low-risk and (D) high-risk group.

Prior to the functional enrichment analysis, we obtained 334 differentially expressed ANGs, and details of these genes are visible in **Table S4**. To clarify the relationship between risk scores and certain biological functions and signaling pathways, we performed GO functional analysis on differentially expressed genes. We used FDR < 0.05 and p < 0.05 conditions to screen for significantly enriched items. According to the results of GO functional analysis **(Figure 8A)**, Biological Processes mainly included cilium organization, cilium assembly, epidermis development, skin development, Cellular Component mainly includes motile cilium, apical part of cell, apical plasma membrane, Cellular Component mainly includes motile cilium, apical part of cell, apical plasma membrane, cytoplasmic region, plasma membrane bounded cell projection cytoplasm, and immunoglobulin complex. However, unfortunately, no significantly enriched items related to Molecular Function were screened accordingly. After GSVA analysis, we found 45 significantly enriched pathways **(Figure 8B, Table S5)**. From GSEA analysis, we obtained pathways active in both high risk and low risk groups **(Figure 8C, D, Table S6)**. Finally, we were surprised by the results of these analyses and found that the results of partial functional enrichment analysis were closely linked to cellular immune responses. To elucidate in detail the immune landscape in the high and low risk groups in CESC patients, we performed a systematic immune correlation analysis of the TCGA-CESC.

### Risk Score Predicts TME and Immune Cell Infifiltration

**Figure 9:**
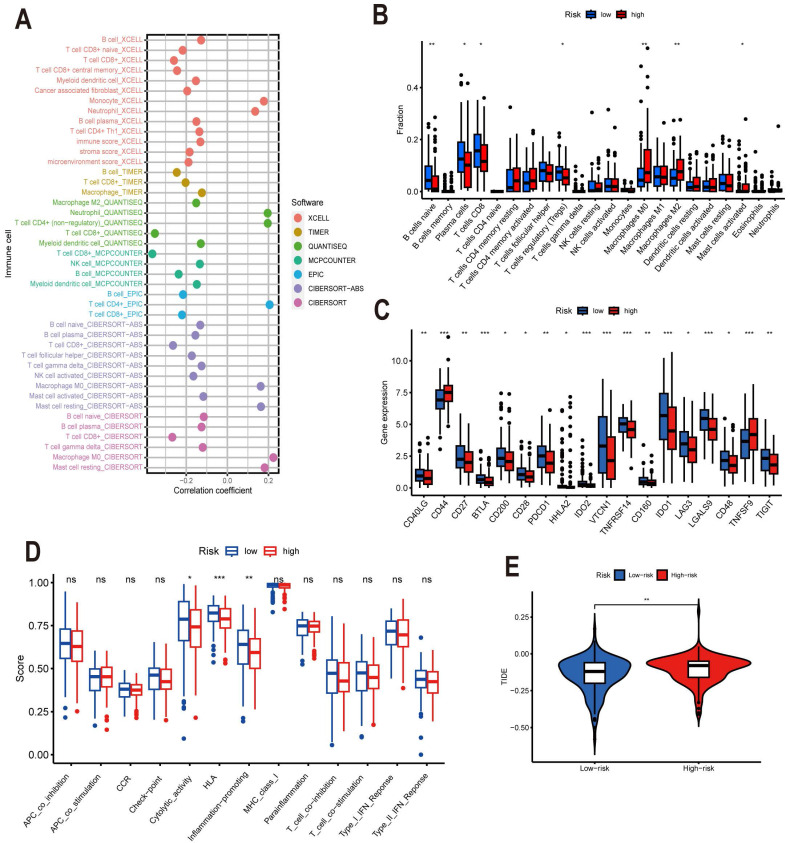
Risk model for ARLs predicts TME and immune cell infiltration. (A) Immune cell bubble map. (B) Discrepancies in immune cell infiltration between high- and low-risk cohorts. (C) Differences in immune checkpoints between high and low risk groups. (D) Immune function ssGSEA scores in the high and low-risk cohorts. (E) Variations in TIDE scores in high and low risk cohorts.

The tumor is infiltrated by multiple immune cells, and these immune infiltrating cells have both tumor-promoting and anti-tumor effects 25. Immunotherapy, a novel and promising therapeutic strategy, is also frequently used in the treatment of cervical cancer, and its therapeutic efficacy is closely related to the immunogenicity of TME. On the basis of this, we conducted a systematic study of immune infiltration and immunological function. We used seven different algorithms, XCELL, TIMER, QUANTISEO, MCPCOUNTER, EPIC, CIBERSORT-ABS, and CIBERSORT, to analyze the correlation between different risk scores and the number of immune infiltrating cells **(Figure 9A)**. Then, in order to compare the immunological infiltration in the high-risk and low-risk groups, we applied the CIBERSORT algorithm. We observed significant distinctions in the expression of B cells naive, Plasma cells, T cells CD8, Tregs, Macrophages M0, Macrophages M2, and Mast cells activated between high and low risk groups **(Figure 9B)**. Based on this, we speculate that the expression and immune activity of these cells may be affected by ARLs, but the shortcoming is that many experimental studies are still required to verify this speculation. Immunotherapy is seen as a promising therapeutic strategy for tumors, and immune checkpoint inhibitors can be used as immunotherapy by reducing the immune escape of tumor cells 26. Immune checkpoints are important for immunotherapy; therefore, we performed an analysis of immune checkpoints between high and low risk groups** (Figure 9C)**. It is notable that only two genes, CD44 and TNFSF9, were notably downregulated in the low-risk group compared to the high-risk group, while CD40LG, CD27, BTLA, CD200, CD28, PDCD1, HHLA2, IDO2, VTCN1, TNFRSF14, CD160, IDO2, LAG3, LAGLS9, CD48, and TIGIT16 genes were significantly upregulated. The expression product of CD44, a non-kinase transmembrane glycoprotein, is a cancer stem cell marker that binds to ligands and induces cell proliferation, increases cell survival and increases cell viability, thereby mediating tumor progression and metastasis 27, 28, 29. It has been shown that TNFSF9 can promote metastasis of pancreatic cancer through Wnt/Snail signaling and macrophage M2 polarization 30. It has also been shown that by targeting TNFSF9 can inhibit the growth and metastasis of prostate cells 31. However, unfortunately, there are no relevant experiments in cervical cancer that can draw similar conclusions as the previous ones. However, based on this we can make a wild guess that low expression of CD44 and TNFSF9 in the low-risk group makes their prognosis better than that of the high-risk group. The risk score combined with patient-specific immune checkpoint gene expression can be used clinically to determine and adjust relevant treatment regimens, thus allowing patients to better benefit from clinical treatment. The above-mentioned studies on immune cell infiltration and immune checkpoints have yielded promising results, but our immune-related studies did not stop there. Since changes in immune cell infiltration usually lead to changes in immune function, we further studied the immune function of CESC patients by ssGSEA analysis. Cytolytic activity, HLA, and inflammation promoting were much lower in the high-risk group compared to the low-risk group **(Figure 9D)**. Finally, we also performed tumor immunosuppression and rejection (TIDE) analysis on the risk model. The implementation of immune checkpoint inhibitor therapy was less successful for patients in the high-risk group due to the TIDE scores in the high-risk group being significantly higher than those in the low-risk group (P<0.05), which indicated a higher chance of immune escape in the high-risk group** (Figure 9E)**.

### Analysis of Tumor Mutation Burden

**Figure 10:**
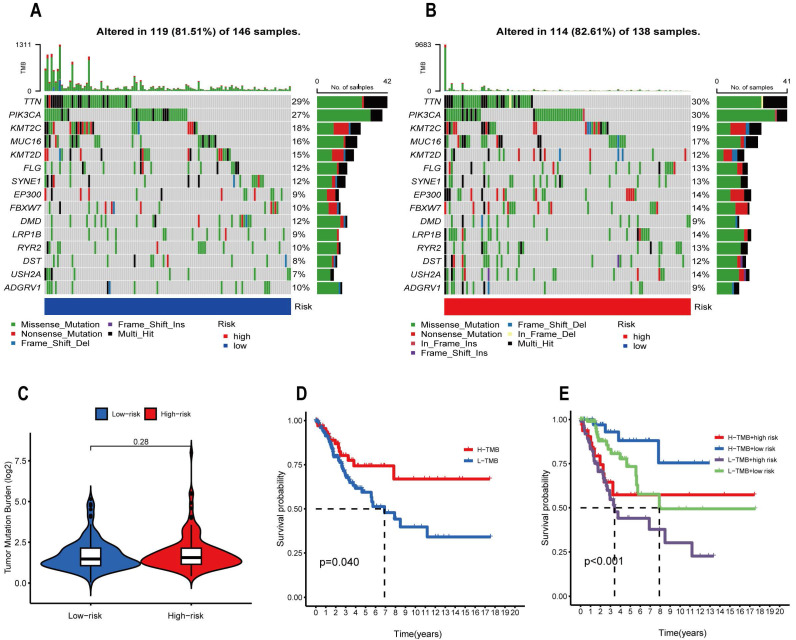
The waterfall plot shows the top 15 genes in the (A) high and (B) low risk groups in terms of mutation frequency. (C) TMB in high and low risk groups. (D) Survival curves of the high and low TMB groups. (E) Survival curves of the high-TMB+high-risk group, high-TMB+low-risk group, low TMB+high-risk group and low-TMB+low-risk group.

TMB represents the number of cancer mutations, with more mutations in cancer cells producing more neoantigens and thus increasing the chance of T cell recognition. Immune checkpoint inhibitor therapy seems to have superior clinical results in patients who have high TMB 32. In the CESC sample, we examined tumor mutations in the high- and low-risk categories. Mutations occurred in 81.5% of the samples in the low-risk group, with TTN (29%), PIK3CA (27%), KMT2C (18%), and MUC16 (16%) being the top four genes with high mutation frequency **(Figure 10A)**. In the high-risk group, 82.61% of the samples were mutated, and the top four genes with high mutation frequency were still TTN (30%), PIK3CA (30%), KMT2C (19%), and MUC16 (17%) **(Figure 10B)**. PIK3CA mutations induce the β-catenin/SIRT3 signaling pathway thereby promoting glycolysis and proliferation of cervical cancer cells, thereby accelerating cancer progression 33. Further research has revealed that patients with positive PIK3CA mutations have lower overall survival when treated with cisplatin. Hence, this population of patients may benefit from PIK3CA inhibitor therapy in conjunction with cisplatin 34. TMB between the low-risk and high-risk groups was compared, and it was discovered that there was no discernible difference between the two groups (P=0.28) **(Figure 10C)**. We also performed an in-depth analysis regarding TMB, dividing CESC patients into low and high mutation groups and performing Kaplan-Meier analysis, and discovered that the high mutation group's survival rate outperformed the low mutation group by a wide margin(p=0.04) **(Figure 10D)**. Finally, we combined the two characteristics of TMB and risk score and divided the sample into four groups, H-TMB+high risk, H-TMB+low risk, L-TMB+high risk, and L-TMB+low risk, and then conducted Kaplan-Meier analysis and found that the survival rate of the H-TMB+low risk group was significantly higher than the other three groups, while the prognosis of L-TMB+high risk group was the worst **(Figure 10E)**. When we use the built model with TMB to forecast a patient's prognosis, we may get a more comprehensive and accurate prediction result.

### Drug Sensitivity Analysis

**Figure 11:**
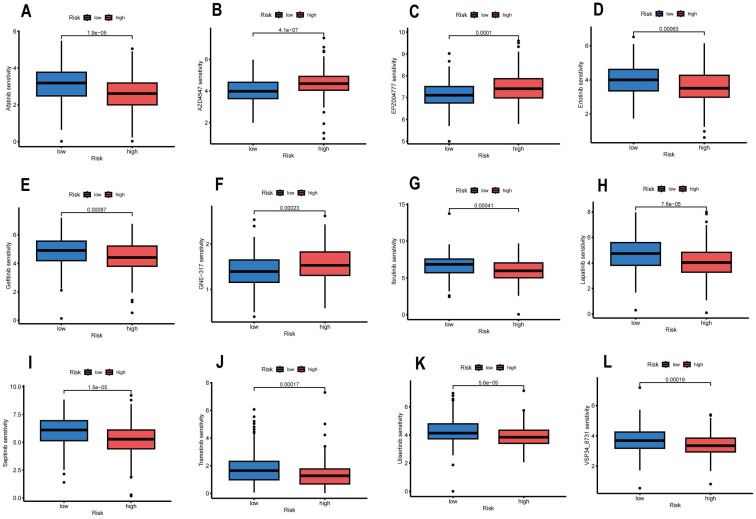
Drug sensitivity analysis in high and low-risk groups. (A) Afatinib, (B) AZD4547, (C) EPZ004777, (D) Erlotinib, (E) Gefitinib, (F) GNE-317, (G) Ibrutinib, (H) Lapatinib, (I) Sapitinib, (J) Trametinib, (K) Ulixertinib, and (L) VSP34_8731.

**Figure 11** shows the 12 popular immunotherapeutic drugs with notable variations in chemosensitivity across individuals at high- and low-risk. We discovered that Afatinib (P=1.9e-05), Erlotinib (P=0.00063), Gefitinib (P=0.00087), Ibrutinib (P=0.00041), Lapatinib (P=7.6e-05), Sapitinib (P=1.5e -05), Trametinib (P=0.00017), Ulixertinib (P=5.8e-05), and VSP34_8731 (P=0.00019) displayed better IC50s in the low-risk group compared to the high-risk group. However, three drugs, AZD4547, EPZ004777, and GNE-317, had high IC50 in the high-risk group. Based on the risk score, we can study the response of CESC patients during immunotherapy in more depth and thus make the drug treatment more precise.

## Discussion

Cervical cancer is the fourth most frequent cancer diagnosis and the fourth dominant cause of cancer-related mortality in women 35. The general outlook for cervical cancer is unfavorable, with a five-year survival rate of only 67%, while the tumor is very prone to recurrence, with about half of patients (44%) having tumor recurrence 36. Treatment of recurrent cervical cancer is extremely challenging, with patients having a five-year survival rate of less than 5% and an extremely poor prognosis 37. Although cervical cancer diagnosis and therapy have made significant strides in recent years, there are still no viable cervical cancer treatments that have been scientifically confirmed to work. Moreover, the prognosis of patients is poor and survival rates have not improved significantly due to tumor recurrence, metastasis, and drug resistance 38. Treatment outcomes and clinical outcomes for patients with advanced cervical cancer are often discouraging. The prognosis of patients is based on their age, tumor status, lymphatic spread, distant metastasis, and histological grading of the tumor, but it is not enough to accurately predict the prognosis based on these indicators 39, 40. International Federation of Late Obstetricians and Gynaecologists (FIGO) staging is a recognized prognostic biomarker for cervical cancer used in clinical practice. It has been suggested that the prognostic validity of FIGO staging should be improved, as survival differences can be observed in patients at the same stage 41. We therefore urgently need to develop more accurate models for risk stratification of patients to predict prognosis, as well as to explore promising prognostic markers. It is commonly acknowledged that angiogenesis significantly contributes to the development and metastasis of tumors 42. It has been shown that angiogenesis in tumors can suppress immune responses and that drugs targeting angiogenesis can stimulate immune responses in the tumor microenvironment thereby enhancing immunotherapy of tumors 43, 44). ARGs have also received a lot of attention. Nevertheless, no research has been done to determine how well ARLs predict outcomes in cervical cancer. We analyzed lncRNA data from CESC samples in the TCGA database to mine prognosis-related lncRNAs and constructed a multi-biomarker prognostic model using ARGs.

In our study, to clarify the potential involvement of ARLs in cervical cancer, we analyzed the lncRNA expression of CESC patients in the TCGA database and employed Lasso and COX regression analysis to identify seven ARLs (MIR210HG, AP001528.1, AC119427.1, AC124045.1, PTPRD-AS1, LINC00683 and KIAA0087) and developed a lncRNA prognostic signature. The analysis revealed that these ARLS are prognostic indicators for cervical cancer and enabled the classification of patients into two distinct subgroups of high and low risk based on risk scores. Further analyses were conducted, and the signatures constructed from these seven ARLs could be seen to have high accuracy for prognosis prediction based on the results of ROC curves and calibration curves. To enhance the accuracy of the model, we also constructed a column line plot by combining the clinical characteristic sexual parameters with the risk score. Finally, we also investigated the correlation between these 7 ARLs in the TCGA-CESC cohort and immune cell infiltration, immunotherapy, tumor mutation, and targeted drug sensitivity analysis. The model constructed based on these seven ARLs may provide new ideas for patient-specific immunotherapy in CESC and provide a theoretical basis for physicians to make clinical decisions that could significantly improve clinical outcomes when patients receive immunotherapy.

LncRNAs have been shown to promote the development of many types of tumors as oncogenic RNAs 45. MIR210HG is a lncRNA induced by hypoxia that promotes cervical cancer 46, gastric cancer 47, glioblastoma multiforme 48, and breast cancer 49 growth and migration, resulting in poor patient prognosis. It has been shown that MIR210HG promotes proliferation, migration, invasion, and epithelial-mesenchymal transition of cervical cancer cells by regulating the miR-503-5p/TRAF4 axis, resulting in tumor growth 46. An additional investigation revealed that MIR210HG is abnormally elevated in cervical cancer and is closely associated with tumor progression, that HPV16 E6/E7 is able to regulate MIR210HG through the transcription factor HIF-1α, and that there is a positive feedback regulation between MIR210HG and HIF-1α. In cervical cancer, phosphoglycerate kinase 1 (PGK1) promotes tumor growth, and MIR210HG may promote PGK1 expression 50. For cervical cancer, the novel prognostic biomarker MIR210HG has unquestionably emerged as a potential therapeutic target and diagnostic biomarker. In one study, prostate cancer tissues showed a substantial down regulation of LINC00683. Survival of patients was closely connected to this lncRNA, with higher levels of its expression associated with better patient prognosis 51. It has been documented that LINC00683 expression is relatively low in recurrent cervical cancer compared to non-recurrent cervical cancer 52. LINC00683 is another intriguing therapeutic target for CESC patients. Based on the available literature we found that AP001528.1, AC119427.1, AC124045.1, PTPRD-AS1, and KIAA0087 could be promising biomarkers for a variety of cancers 53, 54, 55, 56.

The environment in which cancers develop is known as TME. TME is a complex ecosystem of immune cells, fibroblasts, extracellular matrix, and endothelial cells 57. The growth, proliferation, progression, and distant metastasis of cancer are all significantly regulated by TME 58. The processes of angiogenesis promoting cancer growth, invasion, and metastasis are regulated by pro/anti-angiogenic factors secreted by endothelial cells or other cells in TME 59. Studying the function of angiogenesis-related lncRNAs in TME in cervical cancer is essential. TME is highly dynamic and specific, interacting with tumor cells. The composition of TME is one of the important determinants of cancer prognosis and also influences the response of cancer to targeted immunotherapy 60. The immune components are able to suppress immune function and play a crucial role in tumor immune escape 61. Therefore, taking TME into account is one of the elements of a "sound" preclinical prognostic model. To identify probable molecular mechanisms underlying the immune infiltration in TME and to identify novel immunotherapeutic targets to enhance patient prognosis, in-depth research is needed. As a result, we studied samples from high and low-risk groups for immune cell infiltration and immunological function. In anticancer immunotherapy, the use of T cells as the primary immune cells against cancer predominates, however, there is growing evidence that the immunotherapeutic response is strongly affected by B cell-mediated antitumor immunity 62. There is experimental evidence that activated naive B cells can inhibit tumor growth by promoting Th1 polarization 63. In prostate cancer, naive B cells can prevent further development of tumor cells and contribute to a good prognosis for patients 64. Plasma cells, the final functional state of the B cell lineage, are long-lived, non-proliferating, antibody-secreting cells 65. Studies demonstrated a high correlation between immune-infiltrating plasma cells and favorable clinical outcomes and prognosis for patients with a variety of tumor forms 66-68. Our study also corroborates the conclusion that the more significant infiltration of naive B cells and plasma cells in the low-risk group may be associated with a good prognosis of cervical cancer patients. Also, our findings demonstrated that the low-risk group had more activated mast cells expressed than the high-risk group did. Mast cells are derived from CD34+ myeloid precursor cells. Mast cells are a crucial part of the TME in solid tumors and have the ability to either promote or inhibit tumor growth. Whether mast cells inhibit tumor development depends on the nature of the mast cell subpopulation in the TME and the effect of various stimulatory factors on it 69, 70. We also discovered that the major factor connecting mast cells to cancer is their capacity to produce and release strong angiogenic chemicals 71. Nevertheless, the effect of mast cells on cervical cancer has not yet been experimentally validated and the exact mechanism of action still requires extensive studies to elucidate. CD8+ T cells are more infiltrated in the low-risk group, allowing for a better patient prognosis. In cervical cancer, CD8+ T cells—the primary antitumor effector cells—can cause tumor cell death 72, 73. Tregs are widely considered to be a tumor immunosuppressive cell that has a significant impact on the immunological escape process and can make immunotherapy much less effective. In cervical cancer tumor models, it has been discovered that Treg depletion increases the anticancer effectiveness of anti-PD-L1 treatment 74, 75. From the results of our analysis, the low-risk group had better treg infiltration than the high-risk group. This suggests that the administration of anti-PD-L1 therapy to patients in the high-risk group may yield more promising therapeutic results, but this is only speculation based on theory, and it will take a lot of trials to confirm the conclusions.

Immune-targeted therapy for cervical cancer is a promising novel treatment strategy, and the use of immune checkpoint inhibitors (ICIs) in particular has led to improved patient prognosis 76. However, the efficacy of treatment with ICIs in CESC patients is limited, with response rates to ICIs ranging from 10% to 25% 77. Also, the efficacy of this treatment strategy varies from person to person with significant individual differences 78. We should examine how immune checkpoint genes are expressed in our samples and possibly screen for biomarkers that can be used to predict treatment efficacy in order to determine which patients would better benefit from ICIs. This would not only improve the efficiency of treatment but also be enough to reduce the waste of healthcare resources. We discovered that most immune checkpoint gene expression was higher in the low-risk group, indicating that the low-risk group may have better results from ICI treatment. However, this is only a prediction of treatment outcome based on the CESC sample in the TCGA database, and this study used a small sample of data. So to substantiate the findings, many randomized prospective trials are required. If it can successfully identify CESC patients who are well-responding to therapy with ICIs, it will have guiding implications for clinicians' immunotherapy strategies. Immune cells and immunological checkpoints are known to affect immune function, thus we conducted a differential study of immune function. HLA-class I class antigens play a crucial part in neoantigen presentation and cytolytic T cell responses. In contrast, downregulation of the HLA gene decreases antigen presentation and thus contributes to immune escape 79. HLA downregulation has been seen in many cancer types, and it is closely linked to a negative prognosis for the patient 80. HLA expression was shown to be higher in the low-risk group, which similarly suggests that downregulation of the HLA gene is indeed associated with poor clinical outcomes.

Despite the fact that our study had favorable effects on how CESC patients' prognoses are evaluated and guiding clinicians in treatment strategies, there are inevitably certain restrictions. Firstly, further prospective studies are required to verify our model as our work is a retrospective study of data from a public database. Second, the efficacy of our model may be impacted by individual variances in CESC patients. Although we used the TCGA database for internal validation, we weren't able to get accurate lncRNA information due to bias and other limits in the microarray data, which prevented us from using samples from other databases for external validation. Lastly, more in vivo and in vitro studies are required to investigate the relationship between the seven ARLs involved in the modeling and patient prognosis.

## Conclusions

The signature and nomogram constructed in our study displayed accuracy in predicting patient prognosis. This also offers new ideas to mine potential biomarkers for CESC patients. This will not only provide new ideas to explore potential biomarkers for CESC patients but also bring hope to better the patient's prognosis.

## Supplementary Material

Supplementary tables.

## Abbreviations

ARLs: angiogenesis-related lncRNAs
CESC: Cervical squamous cell carcinoma and endocervical adenocarcinoma
lncRNAs: Long non-coding RNAs
ARGs: Angiogenesis-related genes
FDR: false discovery rate
GO: Gene Ontology
TMB: tumor mutation load
IC50: half-maximal inhibitory concentration
GDSC: Genomics of Drug Sensitivity to Cancer
PCA: principal components analysis
BP: Biological Processes
KEGG: Kyoto Encyclopedia of Genes and Genomes
TIDE: tumor immunosuppression and rejection
FIGO: Federation of Late Obstetricians and Gynaecologists
PGK1: phosphoglycerate kinase 1
ICIs: immune checkpoint inhibitors
