# Supplementary material for: Angiogenesis-related lncRNAs index: A predictor for CESC prognosis, immunotherapy efficacy, and chemosensitivity
Source: J Cancer. 2024 Apr 8;15(10):3095–113. doi: 10.7150/jca.94332 (PMC11064265; doi:10.7150/jca.94332)
Supplement: Supplementary file 1 — Supplementary tables. [file jcav15p3095s1.zip › Supplementary Files/Supplementary Table 2.docx]

**Table S2**. Primers used in q-PCR analysis.

| Gene | Sequences (5’-3’) |
| --- | --- |
| MIR210HG Forward | AGGCAGATTTAGTGGACGCC |
| MIR210HG Reverse | ACAGCCTTTCTCAGGTGCAG |
| AP001528.1 Forward | CACCACTGTCAAGCTCACCT |
| AP001528.1 Reverse | GGAGAGCCCAATCAGTCCAG |
| AC119427.1 Forward | GAACCAGTCCGACCAGCTTG |
| AC119427.1 Reverse | AGAGGCTGTTAGATTTTGGGCT |
| AC124045.1 Forward | ACACGGAAGATCAAGCAGCA |
| AC124045.1 Reverse | CTGGGACTACTGGCTTTGCA |
| PTPRD−AS1 Forward | AGCACACAGGAGCTTTGCTA |
| PTPRD−AS1 Reverse | TAGCTTGGCCACTCACTGTG |
| LINC00683 Forward | GGCCTTTTCTCAGGGAGCAT |
| LINC00683 Reverse | CAGGGGCCAAGTGCTCAATA |
| KIAA0087 Forward | AATGGCAGAAGCGGATGTGA |
| KIAA0087 Reverse | CTCAACGGTGTGCAGACTCT |
| GAPDH Forward | CAAAGCCAGAGTCCTTCAGA |
| GAPDH Reverse | CCGCTCATTGCCAATGGTGAT |
